# Long-term trends in Post-COVID severity: a machine learning analysis from the POP/COVIDOM cohort of the German NAPKON Cohort Network

**DOI:** 10.1016/j.eclinm.2026.103822

**Published:** 2026-03-10

**Authors:** Julian Gutzeit, Martin Weiß, Thomas Bahmer, Wolfgang Lieb, Stefan Schreiber, J. Janne Vehreschild, Carolin Nürnberger, Sina M. Pütz, Ekaterina Heim, Anne-Kathrin Ruß, Astrid Dempfle, Michael Krawczak, Susanne Poick, Anna Schäfer, Caroline Morbach, Clara Lehmann, M. Cristina Polidori, Jens-Peter Reese, Thomas Zoller, Lilian Krist, Jan Heyckendorf, Lennart Michel Reinke, Jürgen Deckert, Grit Hein, NAPKON Study Group

**Affiliations:** a Center of Mental Health, Department of Psychiatry, Psychosomatic and Psychotherapy, University Hospital Würzburg, Germany; b Department of Psychology III - Psychological Methods, Cognition, and Applied Research, University of Würzburg, Germany; c Department of Psychology I - Clinical Psychology and Psychotherapy, Julius-Maximilians-Universität Würzburg, Germany; d Internal Medicine Department I, University Hospital Schleswig Holstein Campus Kiel, Germany; e Airway Research Center North (ARCN), German Center for Lung Research (DZL), Grosshansdorf, Germany; f Institute of Epidemiology, Kiel University, Germany; g Department I of Internal Medicine, University of Cologne, Germany; h Division of Infectious Diseases, Department I of Internal Medicine, Faculty of Medicine and University Hospital Cologne, University of Cologne, Germany; i Department II of Internal Medicine, Hematology/Oncology, Goethe University, Frankfurt am Main, Germany; j German Center for Infection Research (DZIF), Partner-Site Cologne-Bonn, Cologne, Germany; k Institute for Clinical Epidemiology and Biometry, University of Würzburg, Germany; l Institute for Medical Data Science, University Hospital Würzburg, Germany; m Trusted Third Party of the University Medicine Greifswald, Germany; n Institute for Medical Informatics and Statistics, Kiel University, Germany; o Department Clinical Research and Epidemiology, Comprehensive Heart Failure Center, University Hospital Würzburg, Germany; p Department of Internal Medicine I, University Hospital Würzburg, Germany; q Center for Molecular Medicine Cologne (CMMC), University of Cologne, Germany; r Ageing Clinical Research, Department II of Internal Medicine and Centre for Molecular Medicine Cologne, University of Cologne Faculty of Medicine and University Hospital Cologne, Germany; s Excellence Cluster CECAD, University of Cologne Faculty of Medicine and University Hospital Cologne, Germany; t Faculty of Health, THM Technische Hochschule Mittelhessen, Giessen, Germany; u Department of Infectious Diseases, Respiratory and Critical Care Medicine, Charité – Universitätsmedizin Berlin, Corporate Member of Freie Universität Berlin and Humboldt-Universität zu Berlin, Germany

**Keywords:** Post-COVID syndrome, Long COVID, Fatigue, Machine-learning, Elastic net regression, Symptom trajectories

## Abstract

**Background:**

Post-COVID syndrome (PCS) affects many survivors with varying symptom profiles driven by acute disease severity (PCS-S) or individual resilience (PCS-R). While cross-sectional studies have identified risk factors and gender differences, long-term trajectories remain unclear. This study investigates the stability and progression of PCS-S and PCS-R scores after 9, 24 and 36 months from initial diagnosis, identifying key predictive factors stratified by gender.

**Methods:**

We analyzed data from 1526 participants of the German National Pandemic Cohort Network (NAPKON), modeling symptom-based PCS-score trajectories over time with linear mixed-effects models. Data were split into training (n = 944), test (n = 233), and two-site external validation (n = 349) sets. Gender-stratified elastic-net regression used nine-month clinical and psychosocial measures to predict PCS scores at 24 and 36 months. All data were collected between November 2020 and February 2024. The study is registered on ClinicalTrials.gov (NCT04679584) and in the German Registry for Clinical Studies (DRKS00023742).

**Findings:**

PCS-S and PCS-R scores showed small but significant declines between 9 and 36 months (β = −0.054 and −0.065, respectively; p < 0.001), indicating persistent symptom burden despite gradual improvement. Predictive models explained 16.7–52.6% of variance in later PCS severity. Fatigue after 9 months and age predicted later PCS-S; quality of life and depression added predictive value in females. Fatigue and sleep issues predicted PCS-R, with living/employment status relevant in females and cognitive deficits in males.

**Interpretation:**

The severity of PCS subtype manifest after 9 months remains relatively stable over time, with distinct gender-specific predictors shaping symptom progression. Tailored interventions are essential for long-term management of PCS pathways.

**Funding:**

The COVIDOM study is funded by the Network University Medicine as part of the NAPKON.

**Research in context**

**Evidence before this study**

The COVIDOM cohort was conceived in March 2020, at a point when almost nothing was known about the long-term consequences of SARS-CoV-2 infection. It was designed to measure the frequency of persistent symptoms and late complications, quantify the lasting health burden, including incident chronic disease, and identify predictors of post-COVID syndrome (PCS).

To inform the present analysis, we conducted an exploratory search of PubMed, Embase, Web of Science and the Cochrane Library for articles published between 1 January 2020 and 29 February 2024, with no language restrictions. Controlled vocabulary and free-text terms combined concepts for “post-COVID syndrome”, “persistent symptoms”, “trajectory”, “predictor” and “longitudinal cohort”. To our knowledge, no previous work produced externally validated, gender-stratified prediction models for distinct PCS sub-phenotypes over a three-year horizon.

**Added value of this study**

Using a population-based German cohort of 1526 participants assessed at 9, 24 and 36 months after infection, we modeled two mechanistically distinct PCS domains: one dominated by acute disease severity (PCS-S) and one driven by individual resilience factors (PCS-R). Elastic-net models trained on 63 clinical, psychosocial and demographic variables collected nine months post-infection explained up to 53% of later variance and retained accuracy in two independent centers. The analysis demonstrates (1) the remarkable stability of symptom burden once established, (2) consistent predictors after 9 months, particularly fatigue and age, and (3) pronounced gender differences, with quality-of-life impairment, depression, social context and work incapacity weighing more heavily in women, whereas cognitive deficits emerge as a key long-term correlate in men.

**Implications of all the available evidence**

The collective evidence indicates that, after nine months, post-COVID symptom burden rarely resolves spontaneously. Symptom-based measures assessed at this stage provide prognostic information for stratifying longer-term symptom trajectories, although predictive accuracy varies by context. From a clinical perspective, this underscores the importance of structured follow-up and symptom-oriented care planning for individuals presenting with high fatigue burden, mental health comorbidities, and sleep disturbance.

## Introduction

Since its emergence in late 2019, COVID-19 has affected hundreds of millions of people worldwide. Despite the shift toward an endemic phase, it continues to present considerable challenges to public health.^1^ Many patients experience long-term postinfectious sequelae—collectively known as long COVID or post-COVID syndrome (PCS)—with reported prevalence ranging from a few percent to over 40%, depending on definitions and populations studied.^2^^,^^3^ This variability reflects the complexity and heterogeneity of PCS, which can involve symptom clusters such as fatigue, cognitive impairment, and respiratory issues.^4^^,^^5^

Recent work on PCS highlights its multifaceted nature and points to distinct symptom constellations within the syndrome. For instance, the PCS score introduced by Bahmer et al.^4^ delineates several symptom complexes and correlates with quality of life, aiding clinical group stratification across healthcare settings.^6^ A recent study further demonstrated that acute COVID-19 severity and individual resilience may differentially shape patients’ post-acute trajectories.^7^ In this study, Ballhausen et al. distinguish two etiological pathways, reflecting the differing influences of acute-phase COVID-19 severity (PCS-S) and personal resilience (PCS-R). The PCS-S score comprises exercise intolerance, fatigue, joint or muscle pain, chemosensory deficits, and signs of infection, collectively defining a “prolonged recovery” subdomain linked to acute-phase severity. In contrast, the PCS-R score encompasses symptom complexes—such as fatigue that persists beyond the acute phase, neurological impairments, and sleep disturbances—primarily shaped by individual resilience. This score classifies patients into two broad groups, marking a “neuro-psychological” PCS phenotype. Together, these novel scores translate patient-reported outcomes into more clearly defined PCS sub-phenotypes, each driven by a distinct etiological pathway.^7^

Emerging evidence also points to gender differences in PCS, with higher prevalence and differing symptom profiles among women.^8^ Women not only tend to experience more severe COVID-19 symptoms^9^ but also exhibit a greater propensity for affective disturbances and depression as part of their post-COVID sequelae.^10^ These findings suggest that resilience and symptom burden may vary by gender, reinforcing the need for gender-stratified analyses of PCS-S and PCS-R.

Concurrently, large-scale cohort efforts like the National Institutes of Health's Researching COVID to Enhance Recovery (RECOVER) Initiative (https://recovercovid.org/) or the German National Pandemic Cohort Network (NAPKON) of the Network University Medicine (NUM^11^) are gathering extensive clinical and self-reported data to better characterize PCS. Recent research has applied machine learning to these datasets to uncover risk factors and symptom patterns, revealing insights often missed by traditional methods (e.g.,^12^, ^13^, ^14^). Our own analysis of NAPKON data, for instance, found substantial overlap between fatigue and depression symptoms, pointing to a strong association.^14^ Other studies have identified pre-infection traits, virus type, vaccination status, and baseline demographics as key predictors of PCS.^12^, ^13^

While large sample sizes in these studies support robust classification, their cross-sectional nature limits insights into PCS progression and recovery markers, critical for successful intervention. Most also rely on binary PCS classifications, overlooking variations in severity.^4^^,^^7^ To address these gaps, our study uses longitudinal data from a German NAPKON cohort^15^ to examine PCS trajectories over 36 months, focusing on how signs of severity and resilience after nine months develop into distinct sub-phenotypes. Using elastic-net regression on data collected nine months post-infection, we predicted PCS-S and PCS-R severity at 24 and 36 months. By integrating clinical and psychosocial factors, we explored whether different PCS subdomains have distinct predictors and may benefit from tailored or unified management strategies.

## Methods

### Sample

Data used for the present analyses stem from the COVIDOM study, which is the population-based cohort platform (POP) of the German National Pandemic Cohort Network (NAPKON) under the Network University Medicine (NUM^11^) and focuses on the long-term sequela of a SARS-CoV-2 infection. The NAPKON study was designed to prospectively recruit approximately 7000 participants between 2020 and 2024, with the sample size determined based on available funding to ensure high-quality data and biospecimen collection across disease strata. Briefly, COVIDOM uses a population-based recruitment strategy in which all PCR-confirmed SARS-CoV-2–infected adults residing in the study regions are identified via local public health authorities that are mandated to register all SARS-CoV-2 infections in their administrative districts. Eligible individuals are identified and contacted by the local health authorities and invited to participate irrespective of acute disease severity, ensuring coverage from asymptomatic to hospitalized cases. After providing informed consent, participants undergo an initial standardized telephone interview and are subsequently invited for a baseline visit at the study center, followed by yearly follow-up assessments. Participants undergo a standardized baseline assessment consisting of structured interviews, validated questionnaires, comprehensive clinical examinations, and biosampling conducted at university hospital study centers. According to the protocol, baseline examinations are scheduled ≥6 months after infection, with follow-up assessments planned at approximately 12–18 months and 24 months. Owing to staggered recruitment and scheduling constraints, the actual examination dates varied across individuals; therefore, in the present analyses we refer to the average realized time points, which corresponded to approximately 9, 24, and 36 months post-infection, rather than the protocol-defined nominal visit windows. Baseline assessments were conducted between November 2020 and June 2022 (median July 2021). More detailed information on the study design, examination procedures, recruitment strategy, and sample size calculation is provided in the COVIDOM study protocol.^11^, ^15^

**Fig. 1:**
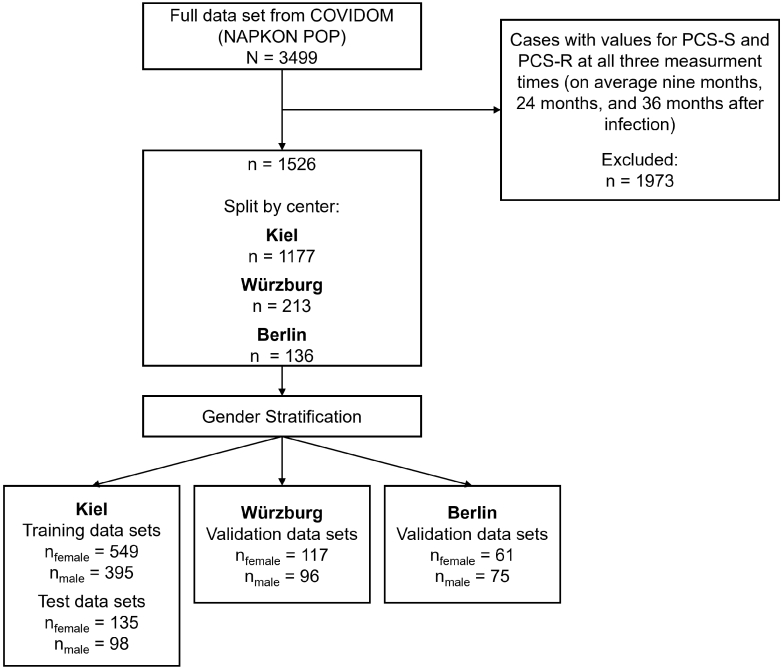
Data retrieval and stratification flowchart.

Participants who provided data at all three measurement times (9, 24, and 36 months post-infection) were recruited from catchment areas surrounding Kiel in northern Germany (n = 1177), Würzburg in Southern Germany (n = 213), and the Neukölln district of Berlin in Eastern Germany (n = 136; see Fig. 1). Eligibility criteria required a positive PCR test for SARS-CoV-2, a minimum interval of nine months between the infection and the baseline COVIDOM examination, and being at least 18 years old at the time of infection. An acute SARS-CoV-2 reinfection at the planned study visit served as the only exclusion criterion. We included participants that provided all the data necessary to compute the PCS-S and PCS-R score.^4^^,^^7^ All data were collected between November 2020 and February 2024.

### Ethics

The COVIDOM study received ethical approval from the local ethics committees of the University Hospitals in Kiel (reference number D 537/20) and Würzburg (reference number 236/20_z). In accordance with the professional code of the Berlin Medical Association, the approval granted by the Kiel ethics committee was also applicable to the Berlin study site. All participants provided written informed consent. The study is registered on ClinicalTrials.gov (NCT04679584) and in the German Registry for Clinical Studies (DRKS00023742).

### Data preprocessing

A total of 131 items were initially selected based on expert knowledge and a review of the literature. These variables were collected at the COVIDOM baseline examination, on average nine months after the initial COVID infection. A threshold of at least 70% valid data in Kiel (complete dataset) was applied in order to retain as many clinically relevant survey items as possible, while still ensuring acceptable data quality. As a result, 69 items with more than 30% missing data were excluded from all data sets. The excluded items mainly reflected granular psychosocial and behavioral domains, including pandemic-related worries, perceived stress and burden, social support and social participation (in-person and digital), detailed physical functioning (PROMIS), work environment and work-related strain, functional limitations, multidimensional fatigue subscales, and selected socioeconomic indicators. Complete missingness patterns for all variables are reported in the Supplementary Materials (Table S1), stratified by study center and gender.

The final set of variables incorporated 62 items encompassing a wide range of variables. These included sociodemographic details such as age, self-reported gender, education, migration background, living situations, and household size. Employment status, changes, and indicators of financial hardship were also considered. COVID-19-related data included information sources, duration of symptoms, and vaccination status. Furthermore, we calculated symptom burden, defined as the total number of existing PCS symptoms, and symptom severity, referring to the total number of severely pronounced PCS symptoms. Additionally, variables linked to health-related quality of life were included, covering perceived health, cognitive functioning (MOCA^16^) and respiratory conditions (mMRC Dyspnea Scale). Psychological and emotional factors such as stress (PSS^17^) general anxiety (GAD-7^18^) depression (PHQ-8^19^) stigma, loneliness (6-ILS^20^) and resilience (BRS^21^) were assessed, alongside COVID-specific evaluations related to chronic fatigue syndrome (FACIT-F^22^) global sleep quality (PSQI^23^) and overall quality of life (EQ-5D-5L^24^) A comprehensive list of all included items is provided in Table S2 of the Supplementary Materials. Importantly, variable inclusion was determined solely on the basis of completion rates in the Kiel dataset. Missingness for some of the retained psychosocial instruments (including FACIT-F, PHQ-8, GAD-7, PSQI, PSS, 6-ILS) was markedly higher in the Würzburg validation cohort, with several of these measures exhibiting very high proportions of missing data (approximately 80–90%) due to their later introduction at that site from mid-2022 onward, resulting in structured, non-random missingness at the 9-month assessment. This site-specific missingness is taken into account in the interpretation of the external validation results.

We then applied an iterative imputation method based on a random forest using the *missForest* package^25^ in R (r core team 2021) on the train data set to obtain a data set without missing values. The obtained prediction parameters were fitted on the test data set and on the validation data sets (see below). By using the imputation parameters from the train data only, we prevented information leakage.

For our outcome variables, we calculated the PCS-S and PCS-R scores as suggested by Ballhausen et al. (2025; see Introduction). In their study, important symptom complexes and their corresponding weights were identified using a Classification and Regression Tree (CART) analysis, which determined the most relevant symptoms for constructing each predictor-specific PCS score. For each symptom complex, it was assessed whether the symptom was present; if so, its corresponding weight was added to the sum score. To ensure that the combined PCS-S and PCS-R scores approximated the original PCS score proposed by Bahmer et al.,(2022) we applied the rescaled weights proposed by Ballhausen et al. Specifically, the weights for PCS-S symptoms were multiplied by 1.048, and those for PCS-R symptoms by 0.124, before rounding to the nearest half-integer. The PCS-S score in this study was calculated by summing the weighted presence of the following symptom complexes: fatigue (weight = 2.5), exercise intolerance (4.5), joint or muscle pain (7.5), chemosensory deficits (6.5), and infection signs (4.0). The PCS-R score included the weighted presence of fatigue (5.5), neurological ailments (5.5), and sleep disturbance (5.5).

### Statistical analyses

To assess the changes of PCS-S and PCS-R scores over time, we fitted a series of linear mixed-effects models using the *lmerTest* package.^26^ Time in months (continuous variable) since the first assessment was grand-mean centered and included as a predictor. Model complexity was increased sequentially by expanding the random-effects structure to account for clustering at the patient and center levels. We first specified a model with random intercepts and slopes for time at the patient level. Next, we tested a nested structure with patients nested within centers, but this model failed to converge. As a final step, we fit a model with random intercepts and slopes for individual patients and an additional random intercept for center. Model fit was compared using maximum likelihood ratio tests, and the best-fitting converging model—the one including random slopes and intercepts for patients plus a random intercept for center—was selected for both PCS-S and PCS-R outcomes.

With the final imputed dataset, collected at the COVIDOM baseline examination, nine months following the acute SARS-CoV-2 infection, we aimed to predict PCS-S and PCS-R scores 24 or 36 months^v^ later, stratified by self-reported gender. For this, we applied elastic net regression, a regularized regression technique. This approach effectively handles multicollinearity among predictors while selecting the most relevant variables. The method regularizes the estimated β coefficients by incorporating a penalty determined by two hyperparameters. The first, α, specifies the type of penalty applied, ranging from ridge regression when α is close to 0 to lasso regression as α approaches 1. The second hyperparameter, λ, controls the degree of penalization, with λ = 0 indicating no shrinkage and increasing λ resulting in progressively greater shrinkage of the coefficients.^27^ This approach is especially well suited for handling high multicollinearity among predictors^27^, ^28^, ^29^ and has previously proven effective in classifying substantial post-COVID sequelae.^14^, ^30^

We used 5-fold cross-validation on 80% of the Kiel dataset (randomly selected; training set) to optimize the hyperparameters α and λ. For each outcome (PCS-S and PCS-R at 24 and 36 months post-infection), stratified by self-reported gender, model selection was based on the highest average R^2^ across folds. The winning model was then refitted on the full training set to derive the final penalized β coefficients. Model performances were then evaluated by applying the models to the remaining 20% of participants from the Kiel center (test dataset) and reporting R^2^, mean absolute error (MAE), and root mean square error (RMSE) for each outcome. We further validated the model performances on two independent validation datasets from patients assessed in Würzburg and Berlin.

To identify the most important predictors, we calculated variable permutation importance scores on the Kiel test data set.^31^ Each variable was permuted 100 times, rendering it effectively non-informative. We refitted the models with the non-informative variable and the resulting decrease in model performance, measured as a reduction in R^2^, was compared to the performance with the original variable. This approach allowed us to estimate each variable's contribution to the model's overall performance. Higher importance scores indicate more predictive value of the variable and thus a stronger association between the variable and the outcome. To investigate the direction and approximate magnitude of associations underlying the predictive models, we performed ordinary least squares (OLS) multiple linear regressions in the Kiel test data set, progressively adding predictors in the order of importance determined by the elastic net regression. The process began with the most important predictor and continued until all predictors were included, ending with the least important. Model selection was based on the Bayesian Information Criterion (BIC) to identify a parsimonious subset of the most informative predictors that best approximated the predictive signal, rather than for formal hypothesis testing; accordingly, p-values from these models should be interpreted descriptively. The model with the lowest BIC was selected as the best-performing one. Finally, we evaluated the top-performing OLS linear regression models with the reduced feature set on the test dataset to assess the generalizability and robustness of our results (for similar approach, see^32^). The analysis code is available at https://osf.io/tzrkn/.

### Role of the funding source

The funder of the study (Network University Medicine, NUM) had no role in study design, data collection, data analysis, data interpretation, or writing of the report.

## Results

### Sample characteristics and data imputation

We applied for the full available POP/COVIDOM dataset at the time of the analyses, which comprised 3499 patients. However, because we were explicitly interested in the time course of PCS over 24 and 36 months, we restricted the sample to participants for whom data from both the second and third follow-up assessments were available, resulting in a final analytic sample of n = 1526 study participants (mean age at nine months after initial Sars-CoV-2 infection *M* = 45.79, *SD* = 15.20, range 18–87; 56.49% female) across all three examination sites. Demographic and clinical characteristics of the study sample at 9 months after infection are depicted in Table 1. The train data sets from the Kiel sample consisted of n = 549 female patients and of n = 395 male patients. The remaining data from the Kiel sample was used as test data set (n = 135 female patients, n = 98 male patients). We also used the data sets from the Berlin (female n = 61, male n = 75) and Würzburg (female n = 117, male n = 96; see Fig. 1) samples as validation data sets. Mean severity of PCS-S and PCS-R across sites and time points is depicted in Table 2. The imputation of the data using *missForest* resulted in an average normalized root mean square error (NRMSE) of 0.767 (SD = 0.232), indicating an acceptable fit.^25^ A Monte Carlo sensitivity analysis demonstrated high stability of the imputation across repeated runs (see Supplement S3).

**Table 1:** Sample size, age, highest educational attainment, migration background, treatment status, and vaccination status at 9 months after SARS-CoV-2 infection by data set and gender.

dataset	gender	n	Age mean (SD)	Proportion with partner	Highest educational attainment			migration background	Proportion treated	Proportion vaccinated
					Lower secondary	Intermediate secondary	Upper secondary			
Kiel (training)	female	548	45.03 (14.74)	77.95%	9.52%	38.83%	47.99%	36.07%	29.20%	56.14%
	male	395	46.53 (15.29)	85.07%	12.44%	29.79%	54.92%	32.00%	27.78%	55.52%
Kiel (test)	female	136	46.57 (13.92)	75.19%	9.02%	37.59%	51.13%	12.50%	33.09%	58.59%
	male	97	50.25 (16.14)	79.12%	19.79%	31.25%	45.83%	14.05%	29.90%	56.38%
Würzburg	female	117	43.85 (17.08)	77.40%	9.43%	16.38%	50.91%	13.40%	16.38%	50.91%
	male	96	46.07 (15.66)	83.91%	13.90%	11.58%	49.46%	15.40%	11.58%	49.46%
Berlin	female	61	43.82 (14.85)	80.70%	11.21%	100.00%	72.13%	12.82%	100.00%	72.13%
	male	75	44.52 (14.83)	87.23%	18.75%	85.71%	68.92%	19.79%	85.71%	68.92%

Note. Educational categories correspond to the German school system: *Lower secondary* includes Mittelschule/Hauptschule; *Intermediate secondary* includes Realschule (or equivalent); and *Upper secondary* includes Fachabitur and Abitur.

**Table 2:** Mean, standard deviation (SD), median, interquartile range (IQR), and range of PCS-S and PCS-R sum scores across study sites and time points (examination cycles), stratified by genders.

outcome	center	months since infection	gender	mean (SD)	median (IQR)	range	n
PCS-R	Kiel	9	female	11.27 (6.21)	16.5 (11)	0–16.5	684
			male	9.58 (6.65)	11 (11)	0–16.5	493
		24	female	9.62 (6.76)	11 (16.5)	0–16.5	684
			male	8.42 (6.79)	11 (16.5)	0–16.5	493
		36	female	9.42 (6.77)	11 (16.5)	0–16.5	684
			male	7.99 (6.87)	5.5 (16.5)	0–16.5	493
	Würzburg	9	female	8.56 (6.79)	11 (16.5)	0–16.5	117
			male	6.76 (7.11)	5.5 (16.5)	0–16.5	96
		24	female	7.57 (6.99)	5.5 (16.5)	0–16.5	117
			male	5.16 (6.72)	0 (11)	0–16.5	96
		36	female	6.63 (6.68)	5.5 (11)	0–16.5	117
			male	5.67 (6.75)	0 (11)	0–16.5	96
	Berlin	9	female	7.75 (7.20)	5.5 (16.5)	0–16.5	61
			male	9.46 (6.56)	11 (11)	0–16.5	75
		24	female	7.93 (6.62)	5.5 (16.5)	0–16.5	61
			male	8.29 (6.97)	11 (16.5)	0–16.5	75
		36	female	7.48 (7.10)	5.5 (16.5)	0–16.5	61
			male	7.85 (6.78)	5.5 (16.5)	0–16.5	75
PCS-S	Kiel	9	female	7.42 (6.68)	7 (11)	0–24.5	684
			male	5.95 (5.86)	6.5 (9)	0–24.5	493
		24	female	6.42 (6.84)	4.5 (11)	0–24.5	684
			male	5.28 (6.12)	2.5 (7)	0–24.5	493
		36	female	5.90 (6.73)	2.5 (11)	0–24.5	684
			male	4.50 (5.84)	2.5 (7)	0–24.5	493
	Würzburg	9	female	4.26 (5.28)	2.5 (6.5)	0–24.5	117
			male	3.20 (5.10)	0 (4.5)	0–24.5	96
		24	female	3.96 (5.42)	0 (7)	0–24.5	117
			male	2.95 (4.51)	0 (6.5)	0–20.5	96
		36	female	2.68 (4.67)	0 (2.5)	0–24.5	117
			male	3.10 (5.01)	0 (6.5)	0–24.5	96
	Berlin	9	female	6.05 (6.53)	6.5 (9)	0–24.5	61
			male	5.29 (5.17)	2.5 (7)	0–18	75
		24	female	4.27 (5.07)	2.5 (7)	0–18	61
			male	3.56 (4.98)	2.5 (6.5)	0–20.5	75
		36	female	4.81 (5.92)	2.5 (7)	0–20.5	61
			male	3.68 (5.61)	2.5 (5.5)	0–24.5	75

### Temporal changes of PCS-scores

**Fig. 2:**
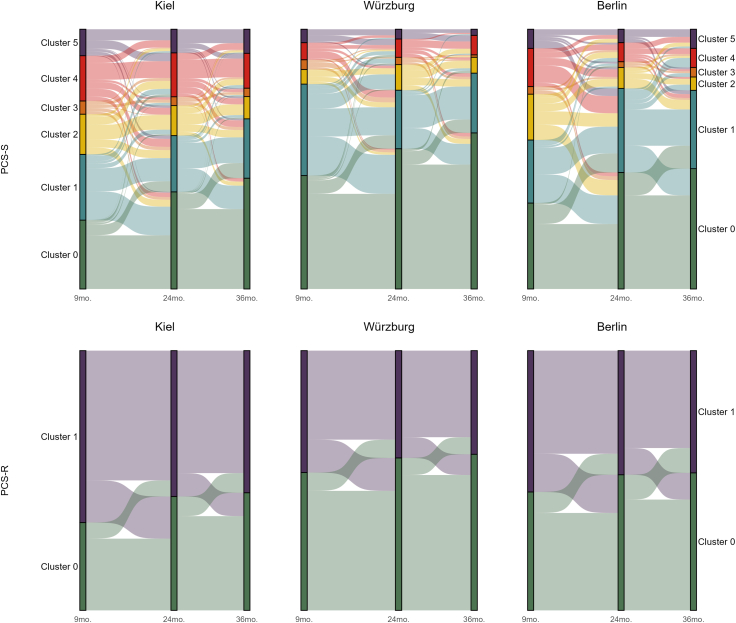
Alluvial plots of relative frequencies of PCS-S (top) and PCS-R (bottom) scores measured at 9, 24, and 36 months post-infection for patients from three centers (Kiel, n = 1177; Würzburg, n = 213; Berlin, n = 136). Clusters were derived from the rescaled scores proposed by Ballhausen et al.,^7^ resulting in six PCS-S clusters (Cluster 0: PCS-S = 0; Cluster 1: 0 < PCS-S ≤ 6.5; Cluster 2: 6.5 < PCS-S ≤ 7.5; Cluster 3: 7.5 < PCS-S ≤ 10; Cluster 4: 10 < PCS-S ≤ 14; Cluster 5: PCS-S > 14) and two PCS-R clusters (Cluster 0: PCS-R ≤ 5.5; Cluster 1: PCS-R > 5.5). The bands indicate how scores change over time, while the rectangles show the relative frequency of each score category.

Linear mixed-effects models were compared to identify the best-fitting structure for modeling post-COVID symptom severity over time. The most complex model, which included a nested random effects structure (patients within centers), failed to converge for both PCS-S and PCS-R outcomes and was therefore excluded. For both PCS-S and PCS-R, the best-fitting model—according to maximum-likelihood ratio test—included random intercepts and slopes of time since infection for individual patients and an additional random intercept for center. Likelihood ratio tests indicated that this model significantly improved fit over a simpler patient-level model without random intercepts for center (PCS-S: χ^2^ (1) = 37.64, p < 0.001; PCS-R: χ^2^ (1) = 33.07, p < 0.001). In the final models, time since initial assessment (i.e., nine months after Sars-CoV-2 infection) was significantly associated with both PCS scores. PCS-S scores decreased over time (β = −0.054, *SE* = 0.005, p < 0.001), as was also observed for PCS-R scores (β = −0.065, *SE* = 0.006, p < 0.001). This suggests that although PCS scores declined significantly over time, the average monthly change was modest—about 0.05–0.07 points per month—amounting to a reduction of roughly 0.6–0.8 points over the course of a year. For both PCS-S and PCS-R, the majority of variance was attributable to between-person differences (PCS-S: τ_00_ = 26.23; PCS-R: τ_00_ = 31.04), with smaller contributions from center-level variation (PCS-S: τ_00_ = 1.78; PCS-R: τ_00_ = 1.91). The intraclass correlation coefficients (PCS-S = 0.76; PCS-R = 0.73) confirmed that most variability occurred between individuals rather than within them. Random slope variances were significant but low (PCS-S: τ_11_ = 0.02; PCS-R: τ_11_ = 0.01), indicating that individual differences in the rate of symptom change over time were modest. Marginal R^2^ values (PCS-S = 0.009; PCS-R = 0.011) showed that time explained only a small fraction of the variance, while conditional R^2^ values (PCS-S = 0.76; PCS-R = 0.74) reflected high overall model fit when accounting for individual and center-level effects. These results indicate that post-COVID scores were highly stable within individuals, with most variance driven by between-person differences and only small, relatively uniform declines associated with time. For visualization purposes, PCS-S and PCS-R scores were grouped into severity clusters based on the rescaled thresholds proposed by Ballhausen et al.^7^ PCS-S was categorized into six clusters reflecting increasing symptom burden: Cluster 0 (PCS-S = 0), Cluster 1 (0 < PCS-S ≤ 6.5), Cluster 2 (6.5 < PCS-S ≤ 7.5), Cluster 3 (7.5 < PCS-S ≤ 10), Cluster 4 (10 < PCS-S ≤ 14), and Cluster 5 (PCS-S > 14). PCS-R scores were dichotomized into Cluster 0 (PCS-R ≤ 5.5) and Cluster 1 (PCS-R > 5.5), indicating lower versus higher resilience-related symptom burden. These clusters were used solely to reduce visual complexity and facilitate interpretation of longitudinal transitions. The fluctuation of clustered PCS-S and PCS-R scores of all patients over time are depicted in Fig. 2.

### Elastic net regression: model performances

#### Prediction of PCS-S scores

The model, based on 63 predictors assessed nine months post-infection, showed moderate to good performance in predicting PCS-S scores in the Kiel test dataset at 24 months (R^2^ = 0.219 for men; R^2^ = 0.501 for women). At 36 months, performance slightly improved for men (R^2^ = 0.275) but declined for women (R^2^ = 0.365). In the Würzburg validation dataset, model performance exceeded that of the Kiel test dataset. At 24 months, R^2^ was highest for men (R^2^ = 0.438) and lower for women (R^2^ = 0.382); at 36 months, it improved for men (R^2^ = 0.462) but declined for women (R^2^ = 0.301). This predictive performance was adequate despite substantial site-specific missingness in several psychosocial predictors (see Methods and Supplementary Materials). In the Berlin dataset, results were more variable: at 24 months, R^2^ values were 0.383 for men and 0.351 for women. At 36 months, Berlin showed the highest performance for women (R^2^ = 0.479), while performance for men dropped to R^2^ = 0.274.

#### Prediction of PCS-R scores

At 24 months, model performance in the Kiel test dataset was strongest for men (R^2^ = 0.511) and slightly lower for women (R^2^ = 0.426). At 36 months, performance declined for men (R^2^ = 0.280) but remained strong for women (R^2^ = 0.519). In the Würzburg validation dataset, predictive accuracy varied: at 24 months, R^2^ was 0.387 for men and 0.304 for women. At 36 months, performance was stable for men (R^2^ = 0.394) but dropped to the lowest level across all sites for women (R^2^ = 0.166) coinciding with extensive site-specific missingness in key psychosocial baseline measures. In the Berlin validation dataset, models performed particularly well for women. At 24 months, R^2^ values were 0.399 for men and 0.478 for women. At 36 months, Berlin showed the highest predictive performance for women across all sites (R^2^ = 0.526), while results for men were moderate (R^2^ = 0.310).

Overall, predictive performance for PCS-S and PCS-R varied across datasets: the PCS-S and PCS-R model performed best on the Kiel test set for men at 24 months, on the Würzburg test set at 36 months, and consistently achieved the highest accuracy on the Berlin test set for women—especially for PCS-R at 36 months. Full performance metrics, including R^2^ and RMSE, are presented in Table 3. Notably, predictive performance in Würzburg remained largely comparable to or higher than that in the Kiel test set despite substantial site-specific missingness in several psychosocial baseline measures, indicating that the models retained external validity under reduced feature availability.

**Table 3:** Performance measures of elastic net regression for PCS-S and PCS-R scores at 24 and 36 months on average after initial Sars-CoV-2 infection for all clinic centers (Kiel, n = 1177; Würzburg, n = 213; Berlin, n = 136).

Outcome	Months since infection	Center	gender	R^2^	RMSE
PCS-S	24	Kiel	female	0.501	5.31
			male	0.219	5.08
		Würzburg	female	0.382	4.45
			male	0.438	3.79
		Berlin	female	0.351	4.22
			male	0.383	4.26
	36	Kiel	female	0.365	5.62
			male	0.275	4.70
		Würzburg	female	0.301	4.37
			male	0.462	3.95
		Berlin	female	0.479	4.43
			male	0.274	4.80
PCS-R	24	Kiel	female	0.426	5.01
			male	0.511	5.01
		Würzburg	female	0.304	6.04
			male	0.387	5.94
		Berlin	female	0.478	5.02
			male	0.399	5.45
	36	Kiel	female	0.519	4.60
			male	0.280	5.83
		Würzburg	female	0.166	6.15
			male	0.394	5.40
		Berlin	female	0.526	5.03
			male	0.310	5.62

Note. RMSE, root mean square error.

### Variable importance

#### Important variables for predicting PCS-S

At 24 months for females, the top five predictors in the model were quality of life (EQ-5D-5L; β = −10.52, p < 0.001),^w^ fatigue (FACIT-F; β = −0.05, p = 0.516), age (β = 0.12, p < 0.001), depression (PHQ-8; β = 0.40, p = 0.044), and symptom burden (β = 0.43, p = 0.001). In this model, fatigue showed no clear association with PCS-S. For males, only fatigue (β = −0.31, p < 0.001) and age (β = 0.10, p = 0.003) emerged as strong predictor of PCS-S scores.

At 36 months, age was no longer among the most relevant predictors of PCS-S scores in females. Instead, quality of life (β = −10.28, p = 0.012), fatigue (β = −0.12, p = 0.039), and symptom severity (β = 0.59, p = 0.001) showed consistent associations with PCS-S, while breathlessness (MMRC; β = −1.82, p = 0.100) did not. Among males, fatigue (β = −0.29, p < 0.001) and age (β = 0.12, p = 0.001) remained strong predictors. Overall, fatigue and age consistently predicted PCS-S across time points, whereas quality of life and symptom burden appeared to be more relevant in females. Full regression coefficients are reported in Table 4. Variance inflation factors (VIFs) indicated no problematic multicollinearity in the PCS-S OLS linear regression models; all predictors had VIFs below 2 for men, and for women only fatigue (VIF = 5.67) and depression (VIF = 5.75) slightly exceeded the conventional threshold of 5 at 24 months, with all VIFs below 2 at 36 months.

**Table 4:** Coefficients of the OLS linear regression on PCS-S with the BIC-selected top predictors of the elastic net regression.

Months since infection	gender	variable	b	SE	t	p
24	female	**QOL (EQ 5D5L)**	**−10.52**	**2.68**	**−3.93**	**<0.001**
		Fatigue (FACIT-F)	−0.05	0.08	−0.65	0.516
		**Age**	**0.12**	**0.03**	**4.09**	**<0.001**
		**Depression (PHQ-8)**	**0.40**	**0.20**	**2.03**	**0.044**
		**Symptom Burden**	**0.43**	**0.12**	**3.47**	**<0.001**
	male	**Fatigue (FACIT-F)**	**−0.31**	**0.06**	**−5.60**	**<0.001**
		**Age**	**0.10**	**0.03**	**3.02**	**0.003**
36	female	**QOL (EQ 5D5L)**	**−10.28**	**4.05**	**−2.54**	**0.012**
		**Fatigue (FACIT-F)**	**−0.12**	**0.06**	**−2.08**	**0.039**
		Breathlessness (MMRC)	−1.82	1.10	−1.66	0.100
		**Symptom Severity**	**0.59**	**0.17**	**3.41**	**<0.001**
	male	**Fatigue (FACIT-F)**	**−0.29**	**0.05**	**−5.95**	**<0.001**
		**Age**	**0.12**	**0.03**	**3.58**	**<0.001**

*Note.* Regression coefficients are shown for the BIC-selected OLS models; p-values are reported for descriptive purposes only and should be interpreted cautiously given the data-driven variable selection. SE, standard error; QOL, quality of life; EQ 5D5L, European Quality of Life 5 Dimensions 5 Level Version; FACIT-F, Functional Assessment of Chronic Illness Therapy—Fatigue; PHQ-8, Patient Health Questionnaire-8; MMRC, modified Medical Research Council Dyspnea Scale. Low Facit-F scores resemble stronger impairment, hence the negative relation. Bold values indicate statistical significance (p < 0.05).

#### Important variables for predicting PCS-R scores

At 24 months, the final PCS-R model for females included fatigue (β = −0.16, p = 0.025), depression (β = 0.12, p = 0.466), sleep quality (β = 0.91, p < 0.001), and symptom burden (β = 0.25, p = 0.022). Fatigue, sleep quality, and symptom burden showed consistent associations with PCS-R, while depression did not. For males, only fatigue (β = −0.50, p < 0.001) was retained, indicating a strong predictive relationship with PCS-R at this time point.

At 36 months, the PCS-R model for females included seven predictors: fatigue (β = −0.16, p = 0.040), depression (β = 0.30, p = 0.066), sleep quality (β = 0.41, p = 0.073), secondary school diploma (β = 1.60, p = 0.065), being unable to work (β = 1.90, p = 0.045), living alone (β = −2.66, p = 0.007), and age (β = 0.06, p = 0.030). Fatigue, inability to work, living alone, and age showed consistent associations with PCS-R, while the others were not. For males, four predictors were retained: fatigue (β = −0.22, p = 0.005), sleep quality (β = 0.56, p = 0.067), cognitive deficits (β = −0.47, p = 0.058), and age (β = 0.09, p = 0.024). Fatigue and age showed consistent associations, while sleep quality and cognitive deficits did not. Full regression coefficients are presented in Table 5. For the PCS-R OLS linear regression models, multicollinearity was generally low; at 24 months all VIFs were below 4, and at 36 months only fatigue (VIF = 4.87) and depression (VIF = 4.20) in women approached the threshold of 5, with all other predictors well below this level.

**Table 5:** Coefficients of the OLS linear regression on PCS-R with the BIC-selected top predictors of the elastic net regression.

Months since infection	gender	variable	β	SE	t	p
26	female	**Fatigue (FACIT-F)**	**−0.16**	**0.07**	**−2.26**	**0.025**
		Depression (PHQ-8)	0.12	0.17	0.73	0.466
		**Sleep Quality (PSQI)**	**0.91**	**0.23**	**4.00**	**<0.001**
		**Symptom Burden**	**0.25**	**0.11**	**2.31**	**0.022**
	male	**Fatigue (FACIT-F)**	**−0.50**	**0.06**	**−8.97**	**<0.001**
36	female	**Fatigue (FACIT-F)**	**−0.16**	**0.08**	**−2.07**	**0.040**
		Depression (PHQ-8)	0.30	0.16	1.86	0.066
		Sleep Quality (PSQI)	0.41	0.23	1.81	0.073
		Secondary School Diploma	1.60	0.86	1.86	0.065
		**Unable to Work**	**1.90**	**0.94**	**2.03**	**0.045**
		**Living Alone**	**−2.66**	**0.97**	**−2.73**	**0.007**
		**Age**	**0.06**	**0.03**	**2.19**	**0.030**
	male	**Fatigue (FACIT-F)**	**−0.22**	**0.08**	**−2.89**	**0.005**
		Sleep Quality (PSQI)	0.56	0.30	1.85	0.067
		Cognitive Deficits (MOCA)	−0.47	0.24	−1.92	0.058
		**Age**	**0.09**	**0.04**	**2.30**	**0.024**

*Note.* Regression coefficients are shown for the BIC-selected OLS models; p-values are reported for descriptive purposes only and should be interpreted cautiously given the data-driven variable selection. SE, standard error; QOL, quality of life; EQ 5D5L, European Quality of Life 5 Dimensions 5 Level Version; FACIT-F, Functional Assessment of Chronic Illness Therapy—Fatigue; PHQ-8, Patient Health Questionnaire-8; MMRC, modified Medical Research Council Dyspnea Scale; PSQI, Pittsburgh Sleep Quality Index; MOCA, Montreal Cognitive Assessment. ‘Secondary School Diploma’ refers to the German *Realschulabschluss*. Low Facit-F scores resemble stronger impairment, hence the negative relation. Bold values indicate statistical significance (p < 0.05).

**Fig. 3:**
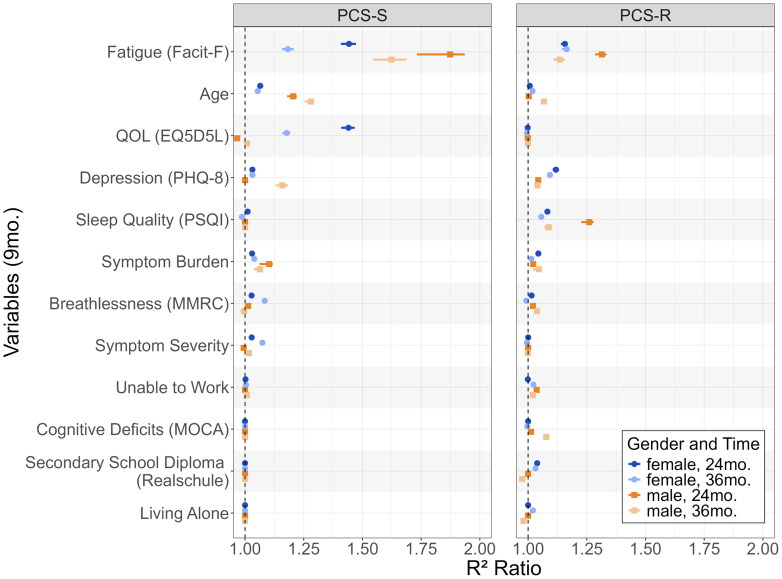
Permutation importance scores (expressed as the ratio of R^2^ reduction when excluding a predictor) for all BIC-selected variables, assessed 9 months after initial SARS-CoV-2 infection. Results are shown for PCS-S (left panel) and PCS-R (right panel) outcomes measured at 24 and 36 months post-infection, stratified by self-reported gender, with 95% confidence intervals.

Fig. 3 depicts the imputation variable importance scores of all variables identified as important in at least one of the models through the BIC selection process.

## Discussion

Using data from 1526 individuals from the German COVIDOM cohort, we assessed the temporal dynamics of two PCS pathways—one linked to acute COVID-19 severity (PCS-S) and the other to individual resilience (PCS-R)—over a 36-month post-infection period and identified relevant gender-specific predictors. Our key findings were: First, both PCS sub-scores after 9 months declined only very gradually, remaining relatively stable throughout the 27-month follow-up period. Second, metrics nine months post-infection proved useful in predicting long-term PCS severity at 24 and 36 months in a gender-differentiated manner. Third, we found that distinct factors influenced PCS outcomes between men and women: in females, poorer quality of life, elevated depressive symptoms, and greater overall symptom burden were more strongly associated with severe PCS-S trajectories. In contrast, fatigue and older age were the predominant predictors in males. Regarding PCS-R, fatigue and sleep disturbances were relevant for males and females; however, for women, additional influences included high symptom load, living alone, and work incapacity, while in men, cognitive impairments were the more prominent predictor.

Importantly, while symptom severity generally declined over the two-year observation period, the rate of improvement was small for both PCS domains—those linked to acute COVID severity (PCS-S) and those more related to personal resilience (PCS-R). Most patients showed little change in their symptoms over time, and differences between individuals remained quite stable. This suggests that once post-COVID symptoms are established, they tend to remain relatively stable, with only modest and uniform improvement in most patients. Importantly, our baseline assessment took place at nine months post-infection, a time point at which PCS can already be considered persistent or chronic. In contrast, longitudinal studies with earlier baseline assessments have reported higher rates of spontaneous remission within the first few months after symptom onset (e.g.,^33^). These differences indicate that once PCS has reached this chronic stage, symptom trajectories appear largely stable^34^^,^^35^ unless targeted interventions are applied. This suggests a critical therapeutic window: by around nine months post-infection, spontaneous remission appears increasingly unlikely, and delaying treatment may reduce the chances of recovery. his underscores the importance of timely, individualized treatment strategies before symptoms become entrenched. Nonetheless, early identification remains challenging due to the lack of standardized definitions and validated diagnostic instruments.^5^

In general, we observed higher PCS-S and PCS-R scores for females compared to males. This finding aligns with recent research demonstrating higher PCS prevalence among women compared to men.^8^

Using elastic-net regression, we predicted PCS severity at 24 and 36 months post-SARS-CoV-2 infection based on data collected nine months after infection. Our models included a wide range of sociodemographic, clinical, and psychological factors, and were trained on the Kiel cohort using a strict train–test split and subsequently evaluated in two independent external validation cohorts (Würzburg and Berlin). Across outcomes, time points, sexes, and centers, predictive performance ranged from moderate to good, explaining 21.9–50.1% of variance for PCS-S and 16.7–52.6% for PCS-R. Given the long prediction horizon of up to 27 months, the heterogeneity of post-COVID symptom profiles, and the reliance on patient-reported severity measures, these levels of explained variance indicate meaningful prognostic signal rather than short-term persistence alone. Unlike prior studies focused on binary classification of PCS status (e.g.,^12^^,^^13^^,^^36^, ^37^), our approach predicts symptom severity along two distinct pathways—one linked to acute COVID severity (PCS-S) and another to resilience factors (PCS-R). This represents a more stringent prediction task than binary classification, as it requires capturing gradations of symptom burden rather than separating cases from non-cases based on threshold definitions. By avoiding dichotomization, continuous severity prediction preserves information on symptom heterogeneity and progression and allows performance to be evaluated using explained-variance and error-based metrics rather than discrimination alone. This continuous, longitudinal modeling captures symptom heterogeneity and progression, offering more precise insights for long-term monitoring and tailored interventions. The robustness of our findings is further underscored by the external validation framework. Although models were trained in a single center, they were evaluated in two independent cohorts with differing case mix, symptom distributions, and data completeness, providing a stringent test of transportability. Despite this, predictive performance consistently remained within the range reported by long-horizon Long-COVID prediction studies with comparable sample size and follow-up duration. For example, Klein et al.^37^ developed a logistic regression model to predict post-COVID condition approximately two years after infection in a cohort of 904 participants and reported a Nagelkerke's pseudo-R^2^ of 0.19 based on in-sample or internally validated classification. While pseudo-R^2^ is not directly comparable to linear-model R^2^, this benchmark illustrates the typical magnitude of predictive performance achieved in long-term post-COVID modeling. When interpreted in absolute terms, lower R^2^ values indicate that a substantial proportion of variance in long-term PCS severity remains unexplained and that predictive accuracy is therefore limited in some strata. However, given the long prediction horizon, the heterogeneity of post-COVID symptom trajectories, and the use of independent external validation, such levels of explained variance are typical for this research context. Viewed in this light, even strata with lower predictive performance in our externally validated severity models fall within this established range, whereas several strata clearly exceed it despite the more demanding continuous outcome and the use of independent external validation. Taken together, these findings support the use of early chronic-phase symptom assessment for context-dependent prognostic stratification of long-term PCS severity rather than universal, site-invariant prediction.

Feature importance analysis revealed key predictors of PCS-S and PCS-R at 24 and 36 months post-infection, based on data collected nine months after initial SARS-CoV-2 infection. For PCS-S at 24 months, lower quality of life, older age, higher depression, and greater symptom burden predicted worse outcomes in females, while fatigue and older age were primary predictors in males. At 36 months, this pattern persisted: in females, poor quality of life, higher fatigue, and symptom severity remained influential, whereas in males, fatigue and age continued to dominate. For PCS-R, fatigue predicted outcomes in both genders at 24 months, but in females, sleep disturbances and symptom burden were also important. Notably, sleep quality (used in PCS-R scoring) emerged as a gender-specific marker of resilience, showing predictive value only in females at 24 (and 36) months.

In line with these findings, recent studies show that women experience sleep disturbances more often than men, typically linked to affective disorders, while in men they are more often tied to respiratory issues.^38^ Thus, sleep disturbances nine months post-infection may indicate lower psychophysiological resilience, particularly in females. Additionally, acute symptom burden predicted PCS-R in women, which is notable since PCS-R reflects stable resilience rather than acute illness severity. However, resilience may also influence symptom burden itself. For example, psychological resilience has been linked to a lower risk of COVID infection,^39^ and personal resilience alongside symptom severity were found to predict early PCS.^4^ Since women tend to experience more severe COVID symptoms,^9^ symptom burden may partly reflect lower resilience in females.

By 36 months after the initial infection, the predictive landscape of PCS-R became more complex, suggesting that the chronic course may depend on more factors than the subacute phase. In females, fatigue at nine months remained a strong predictor of later PCS-R, along with employment status (inability to work linked to higher PCS-R), living alone (associated with lower PCS-R), and age. Employment and living status are known to influence resilience (e.g.,^40^^,^^41^). In males, fatigue, poor sleep quality, cognitive deficits (at trend level), and age were relevant predictors. Since PCS-R captures a neuropsychological PCS profile,^7^ cognitive and sleep disturbances nine months after infection may signal persistent symptom trajectories in men.

Taken together, our analyses suggest that indicators present during the earlier chronic phase of PCS, such as age and fatigue, consistently predict long-term PCS-S severity. Additional factors like reduced quality of life and depressive symptoms appear to play a more prominent role in females. The observed interindividual differences across genders highlight the need for tailored interventions that address specific clinical and demographic risk profiles, with particular attention to the needs of women. For PCS-R, measures of the earlier chronic phase such as fatigue, sleep quality, and symptom burden emerged as key predictors of resilience-related later outcomes, especially in females, while in males, fatigue, sleep quality, and cognitive deficits were influential.

Our finding that chronological age no longer predicted PCS-S severity in women at 36 months, while remaining relevant in men, may reflect a combination of regularization-induced suppression by closely correlated variables (fatigue, health-related quality of life) and a healthy-participant effect: frailer, older women are less likely to attend prolonged follow-ups. This pattern aligns with the well-described ‘Male-Female Health-Survival Paradox’,^42^ whereby women present higher frailty yet maintain better survival than men, possibly masking age-related variance in long-term symptom trajectories.

Interestingly, fatigue in the first nine months after initial infection proved to be an important predictor for the trajectory of COVID-severity related and resilience related PCS. Notably, fatigue predicted PCS-S at 24 months for males but not females. Conversely, depression showed a stronger association with PCS-S outcomes in females than in males. This may reflect the symptom overlap between fatigue and depression,^14^ with females more likely to report depression and males possibly interpreting similar symptoms as fatigue. These findings align with evidence that women are generally more susceptible to depression following COVID-19.^10^

Given that fatigue is a key component in assessing both PCS-S and PCS-R, its presence at earlier stages of the chronic phase likely reflects a relative stability of this symptom—participants experiencing strong fatigue initially continued to be more severely impaired later on, which is in line with recent findings (e.g.,^43^). More generally, several of the predictors identified in our models, including fatigue, sleep quality, breathlessness, and overall symptom severity, overlap conceptually with domains used to construct the PCS-S and PCS-R outcomes. Consequently, their predictive relevance should not be interpreted as evidence for independent causal risk factors, but rather as reflecting persistence and continuity within core PCS symptom dimensions once the chronic phase has been reached. In this context, baseline symptom measures primarily function as prognostic indicators of long-term burden, indicating that individuals with higher symptom severity at approximately nine months post-infection are more likely to remain impaired at later follow-ups. This pattern underscores the relative stability of established PCS symptom profiles and highlights the clinical utility of early chronic-phase symptom assessment for stratifying long-term trajectories, rather than for inferring etiological mechanisms.

This study has several notable strengths. It draws on a large, population-based cohort of 1526 individuals, encompassing a wide range of post-COVID syndrome (PCS) manifestations, which supports the generalizability of the findings. The use of comprehensive and highly standardized clinical assessments at three follow-up time points, extending to 36 months after infection, provides a notably long observational window in PCS research. In addition, the study benefited from uniform data acquisition procedures and the application of advanced machine learning techniques, allowing for a data-driven approach to identify complex patterns and predictors of long-term PCS outcomes. Although the total cohort consisted of roughly 1500 individuals, model development was conducted using sex-stratified datasets, resulting in smaller effective sample sizes for model training (female: n = 548; male: n = 395 and testing (female: n = 136; male: n = 97). Sample size in machine learning must be considered in relation to model complexity rather than in isolation. With 62 predictors and assuming moderate sparsity, the available training sample sizes fall within ranges that prior methodological and simulation studies suggest are generally associated with stable estimation in high-dimensional regression settings.^44^ In the broader Long-COVID research landscape, some studies report substantially larger sample sizes, ranging from multiple tens of thousands (e.g.,^45^) to several million individuals (e.g.,^36^); however, these studies typically rely on routine data sources and lack the extensive longitudinal follow-up available here. Conversely, studies with comparable follow-up durations of 24–36 months after infection generally include only a few hundred participants (e.g.,^46^^,^^47^), placing the present study between these two extremes by combining moderate sample size with deep, long-term phenotyping. We acknowledge, however, that while simulation studies and comparisons with prior empirical research suggest that models of this type and scale tend to exhibit stable behavior, neither simulation evidence nor cross-study comparisons alone can provide definitive proof of stability for the specific models developed here. Nevertheless, our applied modeling strategy followed established gold standards for high-dimensional prediction, including penalized regression with elastic net regularization, rigorous cross-validation, and strict separation of training and test data, thereby maximizing the likelihood of obtaining stable and generalizable model estimates. Importantly, the predictive models were externally validated in two independent datasets from different research sites, further strengthening the robustness of the results. Despite this stringent evaluation framework, predictive performance in the lower-performing strata of our analyses is comparable to, and in several strata exceeds, that reported in recent Long-COVID prediction studies with similar follow-up durations and sample sizes that relied on internal or in-sample validation,^37^ underscoring the robustness of our results.

There are also some limitations. Site-specific missingness of psychosocial predictors, particularly in the Würzburg cohort where several instruments were introduced later, likely attenuated the contribution of fatigue, depression, and sleep measures and contributed to the reduced predictive performance observed for PCS-R in females at 36 months. The preservation of predictive performance under these conditions at the same site in the other cohort or for the prediction of PCS-R at 24 months after infection, however, suggests that the PCS constructs were captured by multiple, partly redundant clinical and demographic indicators, with random forest–based imputation further allowing missing measures to be partially reconstructed from correlated variables.

Additionally, several predictors, such as fatigue, are included as components of the PCS outcome scores. This overlap means that their predictive value may reflect symptom stability rather than independent risk factors, although this remains clinically meaningful given that the PCS scores capture a broad range of symptoms. The presence of certain symptoms at an earlier time point can still inform the expected course of PCS at a later stage in clinical practice. Although information on SARS-CoV-2 reinfections was collected, data completeness was limited and varied substantially across centers and follow-up assessments (see Table S4), precluding reliable inclusion of reinfection status in the analyses. Consequently, we cannot exclude that reinfections may have contributed to symptom persistence, worsening, or the emergence of new symptoms in some individuals. However, an acute reinfection with SARS-CoV-2 at the time of the interview or the scheduled on-site visit constituted an exclusion criterion. Another limitation is the reliance on self-reported data for many variables, which may introduce bias due to recall errors or subjective interpretation.

Finally, the generalizability of the present findings should be interpreted in light of the temporal and structural characteristics of the cohort. Baseline assessments were conducted on average nine months after SARS-CoV-2 infection and during earlier phases of the pandemic (November 2020 to June 2022), implying that most participants were infected between early 2020 and late 2021 and were assessed after symptoms had already persisted for several months. Consequently, the cohort predominantly represents individuals with established, more chronic forms of PCS following infection with the ancestral virus or early variants. Restricting the analyses to participants with complete long-term follow-up may preferentially capture individuals who remained able or willing to engage in repeated assessments and may underrepresent those with rapidly resolving symptoms. Site-specific and structured missingness in psychosocial measures further suggests that certain resilience-related domains may be less comprehensively represented across all centers. Taken together, these factors indicate that the findings are most directly generalizable to individuals with persistent PCS in early-pandemic contexts and should be extrapolated with caution to populations infected with later SARS-CoV-2 variants, under different vaccination conditions, or with transient symptom trajectories. Despite these limitations, the extended follow-up, detailed clinical assessments, population-based recruitment, and rigorous internal and external validation framework strengthen the validity, robustness, and relevance of the study's findings.

Our study demonstrates a marked temporal stability of both the COVID-severity–related (PCS-S) and resilience-related (PCS-R) symptom complexes: individuals who were severely affected at nine months after infection tended to remain similarly impaired at 24 and 36 months, indicating that recovery was generally slow and limited in the absence of specific therapies once PCS had persisted for nine months. Additionally, our findings also highlight distinct gender-specific predictors. In females, lower quality of life, higher depression levels, and greater symptom burden more strongly predicted worse PCS-S outcomes, whereas in males, fatigue and older age emerged as consistent drivers. For PCS-R, fatigue and sleep disturbances played a notable role for both genders, but in females, symptom burden, living status and the inability to work had added relevance, while in males, cognitive deficits was a more influential predictor. These insights underscore the need for individually tailored interventions that account for different trajectories and underlying mechanisms, as well as socio-demographic factors such as employment status and living arrangements as early as possible. A major strength of our work is the use of a robust multi-center design combined with elastic-net machine learning, which allowed us not only to validate our findings across diverse populations but also to go beyond binary classification by predicting severity along two distinct pathways (PCS-S and PCS-R) over 24 and 36 months. This longitudinal, nuanced perspective has important implications for early identification, targeted treatment, and potentially improving long-term outcomes for individuals experiencing chronic PCS.

## Contributors

**Julian Gutzeit:** Conceptualization, Methodology, Software, Validation, Formal Analysis, Data Curation, Writing—Original Draft, Writing—Review & Editing, Visualization. **Martin Weiß:** Conceptualization, Methodology, Software, Validation, Formal Analysis, Data Curation, Writing—Original Draft, Writing—Review & Editing. **Thomas Bahmer:** Conceptualization, Validation, Investigation, Writing—Review & Editing, Project Administration, Funding Acquisition. **Wolfgang Lieb:** Writing—Review & Editing. **Stefan Schreiber:** Writing—Review & Editing. **Jörg Janne Vehreschild:** Writing—Review & Editing.

**Carolin Nürnberger:** Writing—Review & Editing. **Sina M. Pütz:** Writing—Review & Editing. **Ekaterina Heim:** Writing—Review & Editing. **Anne-Kathrin Ruß:** Writing—Review & Editing. **Astrid Dempfle**: Writing—Review & Editing. **Michael Krawcak:** Writing—Review & Editing. **Susanne Poick:** Writing—Review & Editing. **Anna Schäfer:** Writing—Review & Editing. **Caroline Morbach:** Writing—Review & Editing. **Clara Lehmann:** Writing—Review & Editing. **Maria Cristina Polidori:** Writing—Review & Editing. **Jens-Peter Reese:** Writing—Review & Editing. **Thomas Zoller:** Writing—Review & Editing. **Lilian Krist:** Writing—Review & Editing. **Jan Heyckendorf:** Writing—Review & Editing. **Lennart Michel Reinke:** Writing—Review & Editing. **Jürgen Deckert:** Conceptualization, Writing—Review & Editing. **Grit Hein:** Conceptualization, Writing—Original Draft, Writing—Review & Editing, Supervision, Project Administration.

All authors read and approved the final version of the manuscript. Julian Gutzeit and Martin Weiß had access to the data, verified the analyses, and are finally responsible for the decision to submit the current work for publication.

## Data sharing statement

All data of this study may be shared upon request to the NAPKON data use and access committee. Interested parties can access relevant data governance information and submit their research proposal to the NAPKON use and access committee at https://proskive.napkon.de.

## Declaration of generative AI and AI-assisted technologies in the writing process

During the preparation of this work the authors used ChatGPT 4o and o3 (open AI, 2025) in order to improve the readability and language of the manuscript. After using these tools/services, the authors reviewed and edited the content as needed and take full responsibility for the content of the published article.

## Declaration of interests

Thomas Bahmer reports research support from the German Federal Ministry of Education and Research (BMBF) through the Network University Medicine (NUM) for NAPKON (support code 01KX2021). He further reports unrestricted research grants from the German Center for Lung Research (DZL). He received consulting fees from Thieme (editorial board), HealthHero (expert advice), PneumoLounge (podcast), Pohl Boskamp (expert advice), Pfizer (advisory board), Chiesi (advisory board), and AstraZeneca (advisory board). He also received honoraria for lectures from AstraZeneca, Pohl Boskamp, and Pfizer. He serves as General Secretary of the German Society for Pneumology (DGP).

Stefan Schreiber reports consultancy fees from AbbVie, Amgen, Arena, Biogen, BMS, Celgene, Celltrion, Ferring, Fresenius Kabi, Galapagos, Gilead, IMAB, Janssen, Lilly, MSD, Pfizer, Provention Bio, Protagonist, Sandoz/Hexal, Takeda, and Ventyx. He received honoraria for lectures from AbbVie, Alfasigma, Amgen, Arena, Biogen, BMS, Celgene, Celltrion, Falk, Ferring, Fresenius Kabi, Galapagos, Gilead, Hikma, IMAB, Janssen, Lilly, MSD, Pfizer, Provention Bio, Protagonist, Sandoz/Hexal, Takeda, and Ventyx. He also reports travel support from AbbVie, Amgen, Arena, Biogen, BMS, Celgene, Celltrion, Falk, Ferring, Fresenius Kabi, Galapagos, Gilead, IMAB, Janssen, Lilly, MSD, Pfizer, Provention Bio, Protagonist, Sandoz/Hexal, Takeda, and Ventyx.

J. Janne Vehreschild reports grants and contracts from Merck/MSD, Gilead, Pfizer, Astellas Pharma, Basilea, German Centre for Infection Research (DZIF), German Federal Ministry of Education and Research (BMBF), Deutsches Zentrum für Luft-und Raumfahrt (DLR), University of Bristol, Rigshospitalet Copenhagen, German Network University Medicine, German Cancer Consortium (DKTK), German Federal Ministry of Health (BMG), and the European Union. He received honoraria for lectures from Merck/MSD, Gilead, Pfizer, Astellas Pharma, Basilea, DZIF, University Hospital Freiburg/Congress and Communication, Academy for Infectious Medicine, University Manchester, German Society for Infectious Diseases (DGI), Ärztekammer Nordrhein, Ärztekammer Hessen, University Hospital Aachen, Back Bay Strategies, DGIM, Shionogi, Molecular Health, Netzwerk Universitätsmedizin, Janssen, NordForsk, Biontech, APOGEPHA, DKTK, and University Hospital Oldenburg. He reports travel support from DZIF, University Manchester, DGI, University Hospital Aachen, DGIM, Netzwerk Universitätsmedizin, and DKTK. He has served on advisory boards for Merck/MSD, Gilead, Pfizer, Astellas Pharma, Basilea, DZIF, Academy for Infectious Medicine, University Manchester, DGI, DGIM, Netzwerk Universitätsmedizin, Janssen, and Biontech.

Sina Marie Pütz reports honoraria for lectures from Tillotts Pharma GmbH and support for travel from Tillotts Pharma GmbH.

Michael Krawczak reports research support from the German Federal Ministry of Education and Research (BMBF) through the NUM NUKLEUS project (grant no. 01KX2121). He further reports serving as Chairman of TMF—Technology and Methods for Networked Medical Research, Berlin.

Caroline Morbach reports grants/contracts from the Bavarian Ministry of Economic Affairs, Regional Development and Energy, the German Research Foundation, and the Interdisciplinary Center for Clinical Research Würzburg. She received consulting fees and honoraria for lectures from Alnylam, Pfizer, Boehringer Ingelheim, AstraZeneca, NovoNordisk, Alexion, Janssen, Bayer, Eli Lilly, and Bristol Myers Squibb. She further reports travel support from Alnylam, Pfizer, and Bayer.

Clara Lehmann reports grants to her institution from Gilead. She received consulting fees from GSK, Gilead, Biontech, and ViiV. She further reports honoraria for lectures from Gilead, Biontech, Pfizer, and ViiV. She received support for travel from Gilead and has served on advisory boards for Gilead and ViiV.

Jens-Peter Reese reports grants or contracts from the German Ministry of Research and Education within NUM (NPKON, NAPOCO, CAEHR, RECAP) and from the Bavarian State Ministry for Science and the Arts (DigiOnko). He received honoraria from the Landesaerztekammer Hessen for an EBM training lecture. He further reports expert testimony for the German Ministry of Health (BMG).

Thomas Zoller reports funding for the study from the Federal Ministry of Research and Technology of Germany.

Jürgen Deckert reports research support from the German Federal Ministry of Education and Research (BMBF) through the Network University Medicine (NUM) for NAPKON (support code 01KX2021).

All other authors have no conflicts of interest to declare.

## Appendix A Supplementary data

S1 missingness by center and gender

S2 variable overview

S3 sensitivity analysis missForest

S4 reinfection data

## Footnotes

v Note that the time intervals of 24 and 36 months represent average follow-up durations across patients; actual intervals varied slightly between individuals.

w The parameters reported here are from linear regression models in which predictors were sequentially added based on their permutation importance derived from elastic net models.
