# Supplementary material for: Long-term trends in Post-COVID severity: a machine learning analysis from the POP/COVIDOM cohort of the German NAPKON Cohort Network
Source: eClinicalMedicine. 2026 Mar 10;93:103822. doi: 10.1016/j.eclinm.2026.103822 (PMC12995463; doi:10.1016/j.eclinm.2026.103822)
Supplement: S3 sensitivity analysis missForest [file mmc3.docx]

**Sensitivity of missForest imputation to stochastic variation**

We repeated the missForest imputation of the training data 20 times using different random seeds while keeping the train–test split fixed. For females, the normalized mean square error (NMSE) ranged from 0.755 to 0.758 (mean = 0.757, SD = 0.00083), and for males from 0.740 to 0.743 (mean = 0.741, SD = 0.00098). The coefficient of variation was below 0.2% in both groups, indicating negligible imputation variability across runs.

**Table S3.** Results of the sensitivity analysis of the missForest imputation.

| **gender** | **runs** | **NMSE** | | | |
| --- | --- | --- | --- | --- | --- |
|  |  | **mean (*SD*)** | **min** | **max** | **CSV** |
| female | 20 | 0.757 (0.001) | 0.755 | 0.758 | 0.001 |
| male | 20 | 0.741 (0.001) | 0.740 | 0.743 | 0.001 |

Note. NMSE = normalized mean square error, SD = standard deviation, CSV = coefficient of variation (standard deviation divided by mean).
